# Reef structure of the Florida Reef Tract for the period 2005–2020

**DOI:** 10.1007/s10661-023-11819-0

**Published:** 2023-09-22

**Authors:** William S. Fisher

**Affiliations:** Gulf Environmental Measurement and Modeling Division, Office of Research and Development, U.S. Environmental Protection Agency (Emeritus), 1 Sabine Island Drive, Gulf Breeze, FL 32561 USA

**Keywords:** Florida reef tract, Coral size, Coral demographics, Florida reef resilience program, Reef structure

## Abstract

Shallow-water coral reefs of the Florida Reef Tract compose the third largest reef in the world, but during the last several decades, scleractinian (stony) corals have suffered unprecedented declines from global and local stressors. A program to evaluate the effects of high-temperature bleaching events was initiated by The Nature Conservancy’s Florida Reef Resilience Program in 2005 and surveys have been completed across at least some portion of the entire region every year since. The program adopted a demographic (colony-based) assessment approach, which records colony species, size (height and maximum diameter), and estimated partial mortality (percent barren skeleton). Because reef structure is critical to ecosystem functioning and services, data from 2005 to 2020 were analyzed to describe the abundance, size, and morphological complexity of stony coral colonies forming the biogenic reef. Colony height, footprint, surface area, and volume summed for 6016 transects were used to describe reef structure and averages were used to characterize the components that contributed to the structure. Nearly 150,000 colonies representing 49 species were reported during this period and results demonstrated both spatial and temporal changes for the region and for geographic subregions. Some subregions showed increasing colony density, especially for three small, hemispheric species, and declining average colony size.

**Supplementary Information:**

The online version contains supplementary material available at 10.1007/s10661-023-11819-0.

## Introduction

Coral reefs are facing multiple local and global threats to their survival (Eyre et al., 2018; Gardner et al., 2003; Green et al., 2008). Because reefs provide numerous benefits and services to human society, management efforts are continuously underway in the watershed and coastal zone to protect coral reefs from human-generated stressors (Dodge et al., 2008; Fore et al., 2009; Santavy et al., 2022). This requires knowledge and information on the biological condition of reef inhabitants, which are influenced by the cumulative effects of both favorable and unfavorable factors in the environment. Information needs are often met through biological assessments that document current condition and detect change in the status of reef ecosystems. To be effective, assessments measure structural and functional attributes (indicators) that reflect biological or ecological integrity. These often include species composition, diversity, and functional organization (Karr & Chu, 1999) but can also include the condition of key indicator species such as scleractinian (stony) corals. Stony corals are a primary component of the reef structural framework that is integral to many ecosystem functions. They also meet several prerequisites for an effective biological indicator species—they are reasonably abundant, well-distributed, easily identified to species, and not subject to human exploitation (Jameson et al., 1998, 2001).

Some studies have applied a colony-based demographic approach for stony coral biological assessments (Fisher, 2007; Fisher et al., 2007; Ginsburg et al., 1996, 2001; Kramer, 2003; Kramer & Lang, 2003). This approach, in contrast to estimates of two-dimensional coral cover, emphasizes individual organisms as autonomous, self-regulating agents that respond in three dimensions to changing environmental conditions. Species identification, size (diameter and height), and proportion of live or dead tissue are recorded for every colony within a transect. This provides data that can characterize both structural and functional integrity of the population. For example, colony density indicates reproductive success and survival, and the number of species encountered (taxa richness) indicates biological diversity. Large colony size represents long-term supportive environmental quality whereas morbidity (loss of live tissue) can mean near-term or intermittent adverse environmental conditions (Fisher, 2022). Live tissue represents the potential for photosynthesis, calcium carbonate deposition, reproduction, and ultimately population sustainability. Some of these measurements and indicators, including taxa richness, colony surface area, and live colony surface area, have been shown sensitive to human-generated stressors (Fisher et al., 2008; Oliver et al., 2011; Smith et al., 2008) and can be a critical link to management action (e.g., Bradley et al., 2010; Santavy et al., 2022).

Some demographic measurements and indicators provide insight into services and benefits provided to human society (Principe et al., 2012; Yee et al., 2014). Species diversity, density, and colony size can influence site selection for diving and snorkeling tourism (Moberg & Folke, 1999); colony height and surface area provide critical habitat for reef fish that support commercial, artisanal, and subsistence fisheries (Fisher, 2023; Friedlander & Parrish, 1998; Graham & Nash, 2013a, b); and colony height and volume reduce wave energy reaching shorelines to protect property and health (Monismith, 2007; Sheppard et al., 2005). Demographic attributes of stony corals can therefore be useful for evaluating both the ecological integrity of reef ecosystems and the benefits they provide.

Programs that have adopted a demographic survey approach include the Environmental Protection Agency (EPA) to develop Clean Water Act biological water quality standards (Fisher et al., 2014, 2019); components of the National Coral Reef Monitoring Program under aegis of the National Oceanic and Atmospheric Administration (NOAA, 2021); a component of the Coral Reef Evaluation and Monitoring Project (CREMP, 2022); and The Nature Conservancy’s Florida Reef Resilience Program Disturbance Response Monitoring (FRRP, 2023), which is now coordinated through the Florida Fish and Wildlife Conservation Commission with support from EPA’s South Florida Initiative (DRM, 2023). The Disturbance Response Monitoring (DRM) Program has the longest history of continuous surveys and a large dataset of stony coral attributes, including number, species, size, and morbidity (partial mortality). Surveys have been completed annually every autumn since 2005, extending along the entire Florida Reef Tract from Martin County to the Dry Tortugas and covering an area of 251 km^2^ (Smith et al., 2011).

The DRM data are used here to summarize the physical characteristics of stony corals documented through 16 years of the survey (2005–2020). Prior to the survey, reefs in Florida suffered losses from a variety of coral diseases (Dustan & Halas, 1987; Gladfelter, 1982; Kuta & Richardson, 1996), massive sea urchin mortalities (Lessios et al., 1984), and warm temperature events such as occurred during 1982–1983 and 1997–1998 El Niño events (Eakin et al., 2010). Since the survey was initiated, there have been continuing environmental threats to coral condition such as the onset and spread of Stony Coral Tissue Loss Disease (Muller et al., 2020) and multiple hurricanes, including the devastating category 5 Hurricane Irma in 2017. Moreover, temperature events have not subsided (Manzello, 2015) and the summer of 2023 has set thermal high records in waters of the Florida Reef Tract. Previous studies using DRM data have explored homogenization across the reef tract (Burman et al., 2012) and associations with Stony Coral Tissue Loss Disease (Muller et al., 2020).

This study takes advantage of the unprecedented collection of coral demographic data in the DRM dataset to examine several aspects of reef structure and characteristics. Specifically, this study examines colony data—number, size, and complexity—to describe coral reef structure, changes in structure over time and space, and the contributions made by different stony coral species. Reef structure has an important role in reef integrity and ecosystem services, as noted above, and supports the presence and ecological interactions of reef communities (Reaka-Kudla, 1997; Roff et al., 2019; Stella, et al., 2011). Because of these important roles, the loss of reef structure has generated concern (Green et al., 2008; Alvarez-Filip et al., 2009, 2011; Burman et al., 2012; Gonzalez-Barrios & Alvarez-Filip, 2018), and prompted the recommendation by Graham and Nash (2013a, b) that structural complexity becomes an integral goal for reef management and assessment.

## Methods

### Data source

Data analyzed in this study were collected through the Florida Reef Resilience Program’s DRM Program, a project initiated to document stony coral responses to thermal stress (DRM, 2023). Coral condition surveys have been conducted annually from 2005 to 2020 and to the present at shallow-water coral reefs from Martin County to the Dry Tortugas, an ~ 251-km^2^ coastal zone (Smith et al., 2011) often referred to as the Florida Reef Tract. Sampling occurred during a 6- to 8-week period of summer–autumn (Aug–Nov) of each year when thermal stress was at an annual peak and was completed through a collaborative effort of The Nature Conservancy with other non-governmental organizations, academic institutions, and federal, state, and local government agencies (FFWCC, 2022). The survey incorporated a stratified random sampling design whereby non-repeating sites were surveyed within various habitat types and subregions of the reef tract (FFWCC, 2022; Smith et al., 2011).

### Subregion assignments

**Fig. 1:**
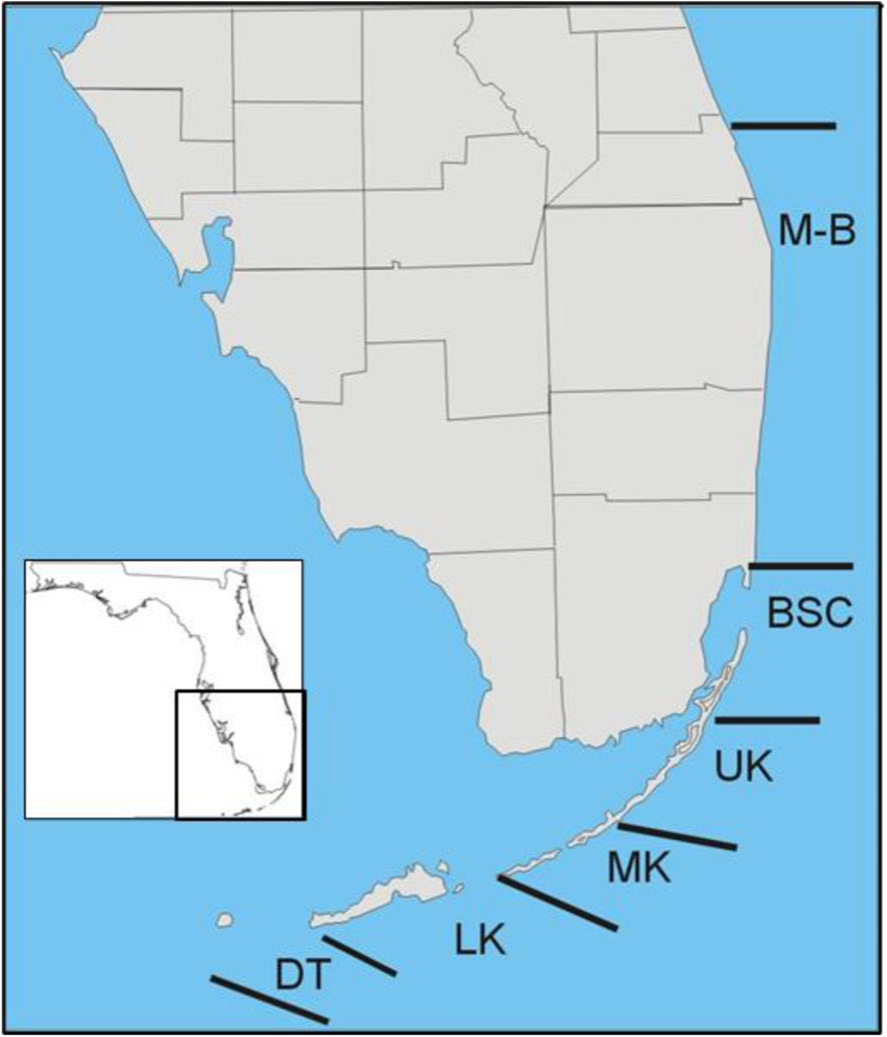
Map of the state of Florida and the Florida Keys Reef Tract divided into geographic subregions Martin-Broward (M-B), Biscayne (BSC), Upper Keys (UK), Middle Keys (MK), Lower Keys (LK), and Marquesas-Dry Tortugas (DT)

For a variety of reasons, including weather and available surveyors, sampling effort was inconsistent and geographic delineations were sometimes re-defined by the program to address different objectives. For this analysis, a 6-subregion geographic approach was adopted to balance as closely as possible the number of colonies in each region (Fig. 1): Results reported for Martin, North Palm Beach, Palm Beach, South Palm Beach, Deerfield, Broward, and Broward-Miami were combined to represent the Martin-Broward (M-B) geographic subregion north of Biscayne Bay; results reported for the Mid-Upper Keys Transition and Middle Keys were combined to represent the Middle Keys (MK) subregion; results reported for Marquesas, Marquesas-Tortugas Transition, Tortugas-Dry Tortugas NP, and Tortugas-Tortugas Bank were combined to represent the Dry Tortugas (DT) subregion; and results reported for Biscayne (BSC), Upper Keys (UK), and Lower Keys (LK) were not combined with any other sampling areas. These six groupings improved consistency in sampling effort across years except for the DT subregion which did not begin until 2007 and was intermittent until 2014 (Table 1).

**Table 1:** The number of transects sampled (#Tr) and coral colonies reported (#Col) for each subregion (northeast to southwest, left to right) during 2005–2020

	**M-B**		**BSC**		**UK**		**MK**		**LK**		**DT**	
**Year**	**#Tr**	**#Col**	**#Tr**	**#Col**	**#Tr**	**#Col**	**#Tr**	**#Col**	**#Tr**	**#Col**	**#Tr**	**#Col**
2005	55	443	22	337	35	400	16	390	50	1402	0	0
2006	60	515	58	1265	56	1108	17	319	38	1171	0	0
2007	66	542	49	1434	47	963	26	604	61	1519	38	464
2008	114	1479	48	1294	79	1344	61	1900	85	3344	0	0
2009	110	955	84	2238	87	1795	47	1414	96	3777	70	1584
2010	103	830	63	1116	87	1947	44	1169	85	2510	0	0
2011	83	863	86	1945	110	2395	61	1960	108	4300	10	123
2012	67	646	62	1440	115	2412	57	1744	67	1874	82	1929
2013	26	330	52	655	42	1053	28	812	41	1199	0	0
2014	78	712	38	563	50	1409	33	745	83	3068	58	1271
2015	171	1463	55	976	46	1368	42	1497	124	3862	40	916
2016	70	485	16	218	26	554	63	1555	79	2580	57	1942
2017	39	207	0	0	0	0	10	504	30	1306	62	1651
2018	89	659	42	1287	46	1346	41	970	58	3063	100	3204
2019	153	1277	30	751	54	1426	54	1370	106	4288	158	7636
2020	218	1710	20	495	70	2120	68	2940	169	6738	216	9173
**Total**	**1502**	**13,116**	**725**	**16,014**	**950**	**21,640**	**668**	**19,893**	**1,280**	**46,001**	**891**	**29,893**

### Annual surveys

Sometimes more than one DRM survey was conducted during a year for alternative purposes such as cold-water events and post-bleaching surveys. Multiple surveys in a single year were identified as separate “batches” using letter designations (DRM, 2023, 2020). For uniformity, this analysis included only one survey for each year of monitoring and did not include any alternative surveys. For years with multiple surveys, the data analyzed were from batches 2006B, 2010C, 2015B, 2016B, and 2017A (DRM, 2023). All other years from 2005 to 2020 had only one survey. Two transects of 1 m × 10 m (10 m^2^ transect area) were surveyed at most sampling locations (sites), although at a few sites 1, 3, or 4 transects were surveyed. For indicator calculations, each transect was treated as a replicate and no transects were combined or averaged to describe site-level characteristics. However, for correlations with latitude, longitude, and depth, the average of co-located transects was used.

### Data exclusions

The original dataset included 6019 transects with records for 154,324 colonies. These numbers were reduced by eliminating colonies of a small size (< 4 cm diameter, as per DRM sampling guidelines) or lacking a measurement dimension (5436 colonies); eliminating colonies identified as “unknown” or only to genus (2073 colonies); and eliminating colonies with 100% mortality (258 colonies). These “standing dead” colonies were excluded because a subjective distinction between old and recent mortalities was required. Remaining for analysis were 146,557 colonies in 6016 transects representing 49 species (Table 1). The distribution of sampling effort across the subregions was M-B 25% of transects; BSC 12%; UK 16%; MK 11%; LK 21%; and DT 15%.

### Colony measurements

The DRM database records the species, width (maximum diameter in planar view), and maximum height (H, maximum dimension perpendicular to the substrate) for all colonies observed in a transect. It also includes estimates of morbidity, i.e., the percent of a coral colony that is lacking live tissue, but those data are not included here.

### Indicator calculations

Colony measurements were adapted from Fisher (2007) to generate stony coral indicators. Colony abundance is reported as density (colonies m^−2^) for each transect; colony size as the sum (Σ) and average (Avg) of colony height (H), footprint (Fp) and volume (Vol); and colony complexity as the Σ and Avg of surface area (SA). Calculating the sum characterizes the reef structure and calculating the average characterizes the colony components of the reef structure. Several similar approaches have been used to estimate coral surfaces based on geometric shapes (Alcala & Vogt, 1997; Bythell et al., 2001; Courtney et al., 2007; Dahl, 1973). A conservative approach used in other assessments (Fisher, 2022) was applied here. Calculations are based on surrogate shapes—a circle for Fp and a hemisphere for SA and Vol. Because colonies are not perfect hemispheres, two modifications to SA calculations were adopted. To account for colony height, r’ was calculated as (r + h)/2. To account for species morphology, a morphological factor (M) was assigned to flat/encrusting (M = 1), hemispheric/massive (M = 2), lobed/domed (M = 3), and branched (M = 4) species. Both height and shape modifications were applied in the hemispheric formula SA = Mπ(r’)^2^. Variable sums were normalized to m^2^ substrate. Standard deviations (sd) are presented to describe variability, and Pearson’s *p*-values are reported for linear regressions, including correlations with latitude, longitude, and depth.

### Statistical applications

Data were compiled and examined for temporal and spatial relationships. Standard deviations are provided for averages. Linear regressions of each variable were examined for significance with Pearson’s *p*-values and confidence intervals (95%) were calculated to demonstrate annual distributions. Subregion data were examined using ANOVA and Tukey’s post hoc test criteria to determine significant differences. Box-and-whisker plots are used to demonstrate the distribution of subregion data.

## Results

### All years and subregions

#### Species distribution

For most annual survey periods, ¾ of the species documented were encountered within the first 100 transects and 90% within 250 transects. Four species, *Siderastrea siderea* (Ssid), *Porites astreoides* (Past), *Stephanocoenia intersepta* (Sint), and *Montastraea cavernosa* (Mcav), were found at over 80%, 70%, 60%, and 50% of the transects, respectively. Among the seven Caribbean/Atlantic scleractinian species listed as threatened by the National Marine Fisheries Service (Federal Register, 2014), *Orbicella faveolata* (Ofav) was most widely distributed (23% of transects), followed by *O. franksi* (Ofra, 8.4%), *O. annularis* (Oann, 4.9%), *Acropora cervicornis* (Acer, 4.3%), *A. palmata* (Apal, 0.3%), *Mycetophyllia ferox* (Mfer, 0.2%), and *Dendrogyra cylindrus* (Dcyl, 0.1%). Among those species highly susceptible to Stony Coral Tissue Loss Disease (Muller et al., 2020; SCTLD, 2018), *Dichocoenia stokesi* (Dsto) was found at 38% of transects, with *Colpophyllia natans* (Cnat) and *Meandrina meandrites* (Mmea) at 16–17% of transects.

#### Density

Avg density across the survey was 2.44 colonies m^−2^ (sd = 2.44, Table 2), varying across survey years from 1.67 to 3.05 and across subregions from 0.88 to 3.60 colonies m^−2^. Species distributions across subregions are presented in SI-1. The highest density for any transect was 24 colonies m^−2^ which occurred at LK in 2018. Highest densities were recorded for Ssid, Past, Sint, and Mcav. Among threatened species, Ofav had the highest density followed by Ofra, Oann, and Acer, while Apal, Dcyl, and Mfer were all < 0.001 colonies m^−2^. Additional data on the status of threatened species is available at NOAA (2014a, b).

**Table 2:** Colony density (n m^−2^) of species recorded in DRM surveys (2005–2020) and proportion (%) of the total population. Acronyms used in the text are shown for each species. A list of species by subregion is provided in online resource SI-1

**Species**	**Acronym**	**Density** n m^−2^	**%**	**Species**	**Acronym**	**Density** n m^−2^	**%**
*Acropora cervicornis*	Acer	0.0134	0.55	*Meandrina meandrites*	Mmea	0.0226	0.93
*Acropora palmata*	Apal	0.0007	0.03	*Montastraea cavernosa*	Mcav	0.1779	7.30
*Acropora prolifera*	Apro	0.0001	0.01	*Mussa angulosa*	Mang	0.0026	0.11
*Agaricia agaricites*	Aaga	0.1274	5.23	*Mycetophyllia aliciae*	Mali	0.0023	0.09
*Agaricia fragilis*	Afra	0.0021	0.09	*Mycetophyllia ferox*	Mfer	0.0003	0.01
*Agaricia grahame*	Agra	0.0000	0.00	*Mycetophyllia lamarckiana*	Mlam	0.0005	0.02
*Agaricia humilis*	Ahum	0.0034	0.14	*Oculina diffusa*	Odif	0.0041	0.17
*Agaricia lamarcki*	Alam	0.0074	0.30	*Orbicella annularis*	Oann	0.0132	0.54
*Agaricia tenuifolia*	Aten	0.0000	0.00	*Orbicella faveolata*	Ofav	0.0491	2.01
*Cladocera arbuscula*	Carb	0.0004	0.02	*Orbicella franksi*	Ofra	0.0218	0.90
*Colpophyllia natans*	Cnat	0.0390	1.60	*Porites astreoides*	Past	0.4138	16.99
*Dendrogyra cylindrus*	Dcyl	0.0001	0.01	*Porites branneri*	Pbra	0.0032	0.13
*Dichocoenia stokesi*	Dsto	0.0729	2.99	*Porites divaricata*	Pdiv	0.0277	1.14
*Diploria labyrinthiformis*	Dlab	0.0135	0.55	*Porites furcata*	Pfur	0.0229	0.94
*Eusmilia fastigiata*	Efas	0.0148	0.61	*Porites porites*	Ppor	0.1217	5.00
*Favia fragum*	Ffra	0.0018	0.07	*Pseudodiploria clivosa*	Pcli	0.0129	0.53
*Helioceris cucullata*	Hcuc	0.0020	0.08	*Pseudodiploria strigosa*	Pstr	0.0283	1.16
*Isophyllastrea rigida*	Irig	0.0002	0.01	*Scolymia cubensis*	Scub	0.0006	0.03
*Isophyllia sinuosa*	Isin	0.0008	0.03	*Scolymia lacera*	Slac	0.0000	0.00
*Madracis auretenra*	Maur	0.0045	0.18	*Siderastrea radians*	Srad	0.0659	2.70
*Madracis decactis*	Mdec	0.0140	0.58	*Siderastrea siderea*	Ssid	0.7691	31.57
*Madracis formosa*	Mfor	0.0008	0.03	*Solenastrea bournoni*	Sbou	0.0305	1.25
*Madricis senaria*	Msen	0.0001	0.00	*Solenastrea hyades*	Shya	0.0027	0.11
*Mancina areolata*	Mare	0.0025	0.10	*Stephanocoenia intersepta*	Sint	0.3202	13.14
*Meandrina jacksoni*	Mjac	0.0000	0.00	**All species**		**2.4361**	**100**

#### Colony height

The sum of heights (ΣH; Table 3) across all transects and species averaged 20.7 cm m^−2^ (sd = 25.0), with Ssid (5.8 cm m^−2^), Mcav (3.1 cm m^−2^), and Past (2.2 cm m^−2^) contributing the most among species. Average height (Avg H) was 8.5 cm colony^−1^ (range 1–500 cm; sd = 12.2). Tallest Avg H was for Oann (38.0 cm), Ofav (36.9 cm), Apal (31.3 cm), Dcyl (27.1 cm), and Ofra (23.0 cm). Overall, 20% of colonies (range 15–26% across survey years) were > 10 cm tall (H_>10_), contributing 58% of the total H (range 54–66% across survey years). Colonies > 50 cm tall (H_>50_) comprised 1.3% of the recorded population and those > 100 cm tall (H_>100_) comprised 0.2%. Over 2/3 ${}^{2}/_{3}$ (69%) of H_>100_ colonies were orbicellids (Oann, Ofav, and Ofra) followed by Mcav (16%). Only three acroporid colonies, all Apal, were among the H_>100_ colonies reported.

**Table 3:** Averages, ranges and standard deviations (sd) for size and complexity measurements on coral colonies, including data for the sum of each variable m^−2^ and the average of each variable colony^−1^. Shown are colony height (H, cm), footprint (Fp, cm^2^), volume (Vol, cm^3^), and surface area (SA, cm^2^) for all years and all subregions combined. The ranges represent variables from transects and colonies with the highest and lowest values

Variable	Sum m^−2^	Range	sd	Avg colony^−1^	Range	sd
Colony height (H)	20.7	0.1–353	25.0	8.49	1–500	12.2
Colony footprint (Fp)	1223	1–41,562	2500	502	1–159,043	2375
Colony volume (Vol)	39,081	1–8,829,495	194,093	16,042	3–41,887,901	256,479
Colony surface area (SA)	2897	1–228,977	6853	1189	10–849,230	6985

#### Colony footprint

The sum of colony footprint (ΣFp) across transects and species was 1223 cm^2^ m^−2^ (sd = 2500; Table 3) and the greatest contributions were from Ssid (297 cm^2^ m^−2^), Mcav (224 cm^2^ m^−2^), and Ofav (206 cm^2^ m^−2^) colonies. Avg Fp was 502 cm^2^ colony^−1^ (range 0.8–159,043; sd = 2375) and species with the largest Avg Fp were Oann (5–384 cm^2^), Ofav (4208 cm^2^), Apal (3879 cm^2^), Cnat (2378 cm^2^), and Ofra (2247 cm^2^).

#### Colony volume

The sum of colony volume (ΣVol) across transects and species averaged 39,081 cm^3^ m^−2^ (sd = 194,093; Table 3) and the largest volume summed for any transect was 8,829,497 cm^3^ m^−2^, which occurred at LK in 2016. The largest contributions were from Ofav (12,436 cm^3^ m^−2^), Mcav (7323 cm^3^ m^−2^), Ssid (5691 cm^3^ m^−2^), and Oann (5302 cm^3^ m^−2^). Avg Vol was 16,042 cm^3^ colony^−1^ (range 2.6–41,887,902; sd = 256,479) and the highest Avg Vol was for Oann (401,755 cm^3^), Ofav (253,435 cm^3^), Apal (233,798 cm^3^), and Dcyl (130,582 cm^3^).

#### Complexity

The sum of surface area (ΣSA) across transects and species was 2897 cm^2^ m^−2^ (sd = 6853; Table 3), with the greatest contributions from Ssid (601 cm^2^ m^−2^), Ofav (561 cm^2^ m^−2^), and Mcav (548 cm^2^ m^−2^). Avg SA was 1189 cm^2^ colony^−1^ (range 9.6–848,230; sd = 6985). Largest Avg SA were for Apal (21,470 cm^2^), Oann (20,660 cm^2^), and Dcyl (12,918 cm^2^).

### Spatial and temporal variation

#### Density

**Fig. 2:**
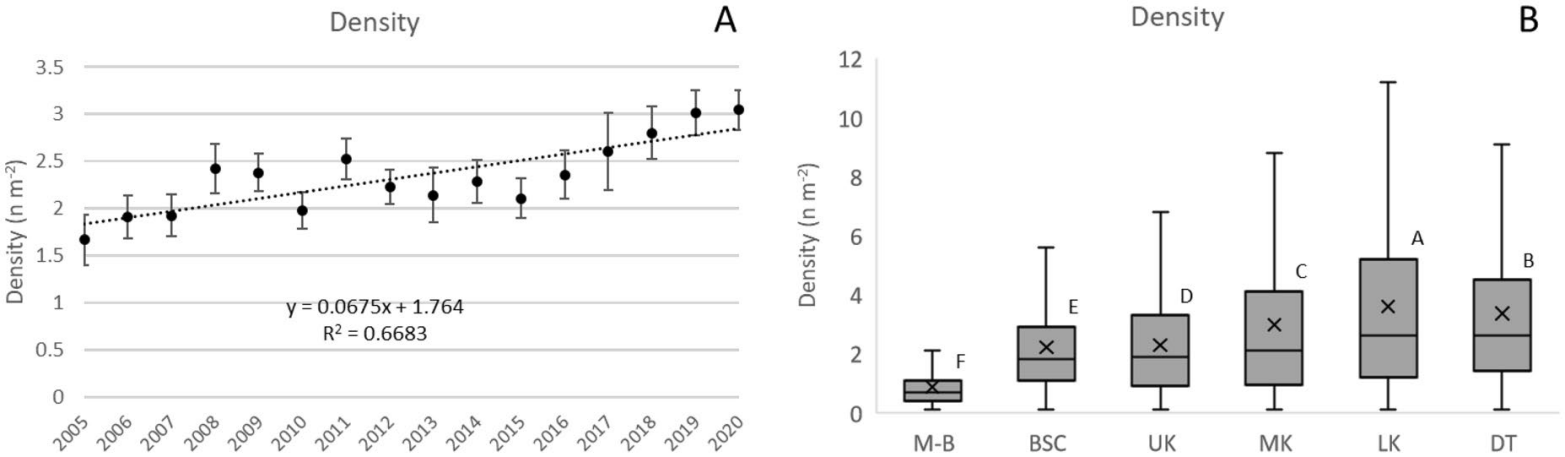
**A** Avg density (n m^−2^) of colonies recorded from 2005 to 2020 at all subregions increased 0.068 colonies m^−2^ year^−1^ (*p* < 0.001). Vertical bars are 95% confidence intervals for each annual average. **B** Average density of colonies showed significant differences among subregions determined through analysis of variance using Tukey’s post hoc test criteria (letter designations, *p* < 0.05). Boxplots show mean (x), median (crossbar) and data quartiles. Letters designate differences (*p <* 0.05) across subregions using analysis of variance and Tukey’s *post hoc* test criteria

Avg density across the Florida Keys increased from 1.66 in 2005 to 3.05 colonies m^−2^ in 2020, or 0.068 colonies m^−2^ year^−1^ (*p* < 0.001; Fig. 2A). This change was largely attributable to increased densities of Ssid (0.03 m^−2^ year^−1^, *p* < 0.001), Sint (0.02 m^−2^ year^−1^, *p* < 0.001), and Past (0.01 m^−2^ year^−1^, *p* < 0.01). Median density increased from 1.1 in 2005 to 2.1 colonies m^−2^ in 2020, or 0.045 colonies m^−2^ year^−1^ (*p* < 0.01). Ssid comprised the highest proportion of colonies for each survey period (average 31.4%) and this proportion increased from 22.2% in 2005 to 36.7% in 2020 (0.74% year^−1^, *p* < 0.001). The proportion of Sint increased 0.28% year^−1^ (*p* < 0.01) but the proportion of Past did not change significantly (*p* > 0.05). There was no significant change in density for Ofav and Mcav. Subregional analysis showed subregional differences with LK having the highest density and M-B the lowest (Fig. 2B).

#### Colony height

**Fig. 3:**
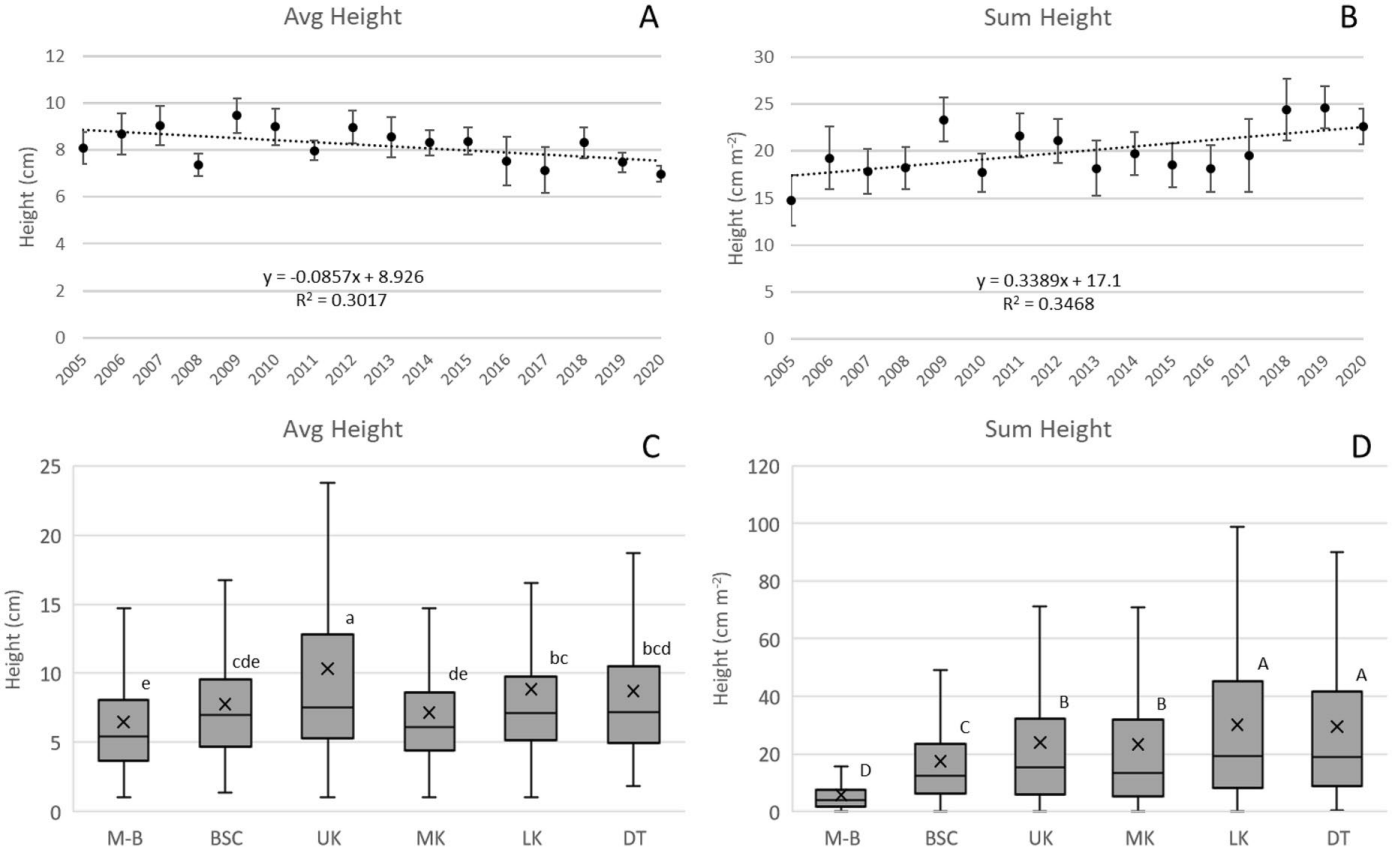
**A** Change over the duration of the survey (2005–2020) in Avg H, which declined 0.09 cm year^−1^ (*p* < 0.05). **B** Change in ΣH during the survey, which increased 0.34 cm m^−2^ year^−1^ (*p* < 0.05). Vertical bars are 95% confidence intervals for each annual average. **C** and **D** Differences in Avg H and ΣH across subregions. Boxplots show mean (x), median (crossbar) and data quartiles. Letters designate differences (*p* < 0.05) across subregions determined through analysis of variance using Tukey’s *post hoc *test criteria

Colony heights were largely in the 0–10 cm range (78%) with over 95% of colonies < 30 cm height. The proportion of H_>10_ colonies decreased by 0.5% year^−1^ (*p* < 0.01) but average density of H_>10_ colonies, which ranged from 0.35 to 0.62 colonies m^−2^, did not significantly change (*p* > 0.05). There were no changes in H_>50_ density and a small increase for H_>100_ density (0.0003 colonies m^−2^, *p* < 0.05). Avg H declined 0.09 cm year^−1^ (*p* < 0.05) during the 2005 − 2020 survey period (Fig. 3A), much of which can be attributed to declines in Ssid (0.15 cm year^−1^, *p* < 0.001), Past (0.08 cm year^−1^, *p* < 0.01), and Sint (0.08 cm year^−1^, *p* < 0.01). Nonetheless, ΣH increased by 0.34 cm m^−2^ year^−1^ (*p* < 0.05; Fig. 3B). On average, the tallest colonies were at UK (Fig. 3C), but higher densities generated greater ΣH at LK and DT (Fig. 3D).

#### Colony footprint

**Fig. 4:**
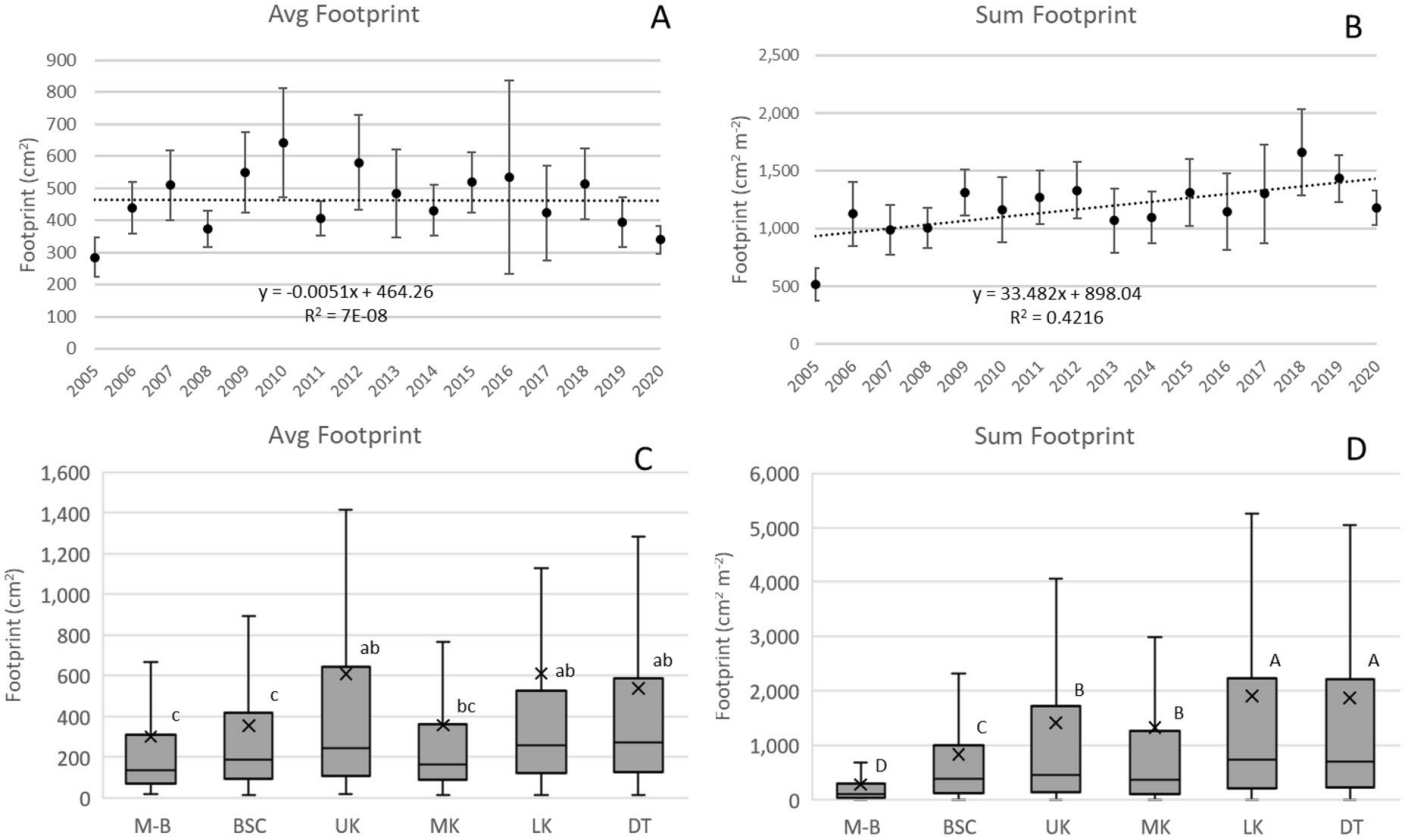
**A** and **B** Change over the duration of the survey (2005–2020) in average Fp and ΣFp, the latter which increased 33.5 cm^2^ m^−2^ year^−1^ (*p* < 0.05). Vertical bars are 95% confidence intervals for each annual average. **C** and **D** Differences in average and ΣFp across subregions. Boxplots show mean (x), median (crossbar) and data quartiles. Letters designate differences (*p* < 0.05) across subregions determined through analysis of variance using Tukey’s *post hoc* test criteria

Avg Fp did not significantly change during the survey period (Fig. 4A), but ΣFp increased 33.5 cm^2^ m^−2^ year^−1^ (*p* < 0.01, Fig. 4B) driven largely by Ssid, Mcav, and Ofav, which increased a combined 27.9 cm^2^ m^−2^ year^−1^ (*p* < 0.01). Average colony Fp was smallest at M-B and largest at UK, LK, and DT (Fig. 4C), and ΣFp was likewise less at M-B and more at LK and DT (Fig. 4D). One transect surveyed at LK in 2016 could be considered an outlier; there were nine colonies in the transect, including 3 Oann with 400 cm width (Fp = 125,664 cm^2^ each) and 1 Oann each at 200 cm (Fp = 31,415), 75 cm (Fp = 4418), and 50 cm (Fp = 1963) width. This transect generated the high confidence intervals (CI) shown for 2016 (Fig. 4A), and if the transect is eliminated, the CI shown is reduced by about half.

#### Colony surface area

**Fig. 5:**
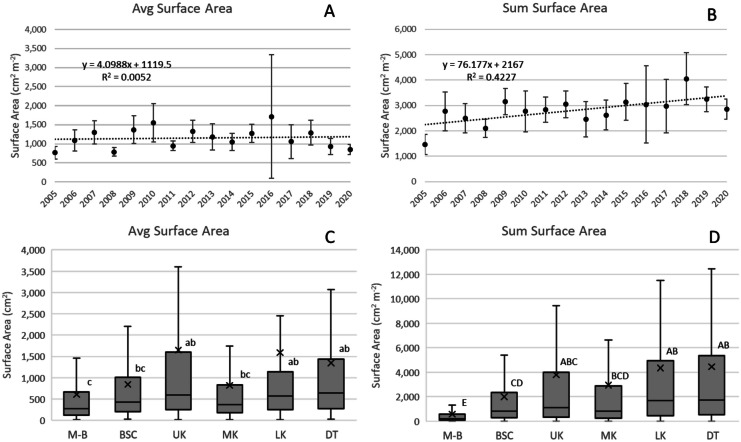
**A** and **B** Change over the duration of the survey (2005–2020) in average SA and ΣSA, the latter increasing 76.2 cm^2^ m^−2^ year^−1^ (*p* < 0.01). Vertical bars are 95% confidence intervals for each annual average. **C** and **D** Differences in average and ΣSA across subregions. Boxplots show mean (x), median (crossbar) and data quartiles. Letters designate differences (*p* < 0.05) across subregions determined through analysis of variance using Tukey’s *post hoc* test criteria

There was no significant temporal change in Avg SA (Fig. 5A), but ΣSA increased 76.2 cm^2^ m^−2^ year^−1^ (*p* < 0.01, Fig. 5B). Ssid, Ofav, and Mcav combined for most of this increase (64.6 cm^2^ m^−2^ year^−1^; *p* < 0.01). Average SA was smallest at M-B and largest at UK, LK, and DT (Fig. 5C); ΣSA was similarly less at M-B and greater at UK and DT (Fig. 5D). One transect surveyed at LK in 2016 could be considered an outlier; there were nine colonies in the transect, including 4 Oann with SA between 212,000 and 848,000 cm^2^. This transect generated the high CI shown for 2016 (Fig. 5A), and if the transect is eliminated the CI shown is reduced by about half.

#### Colony volume

**Fig. 6:**
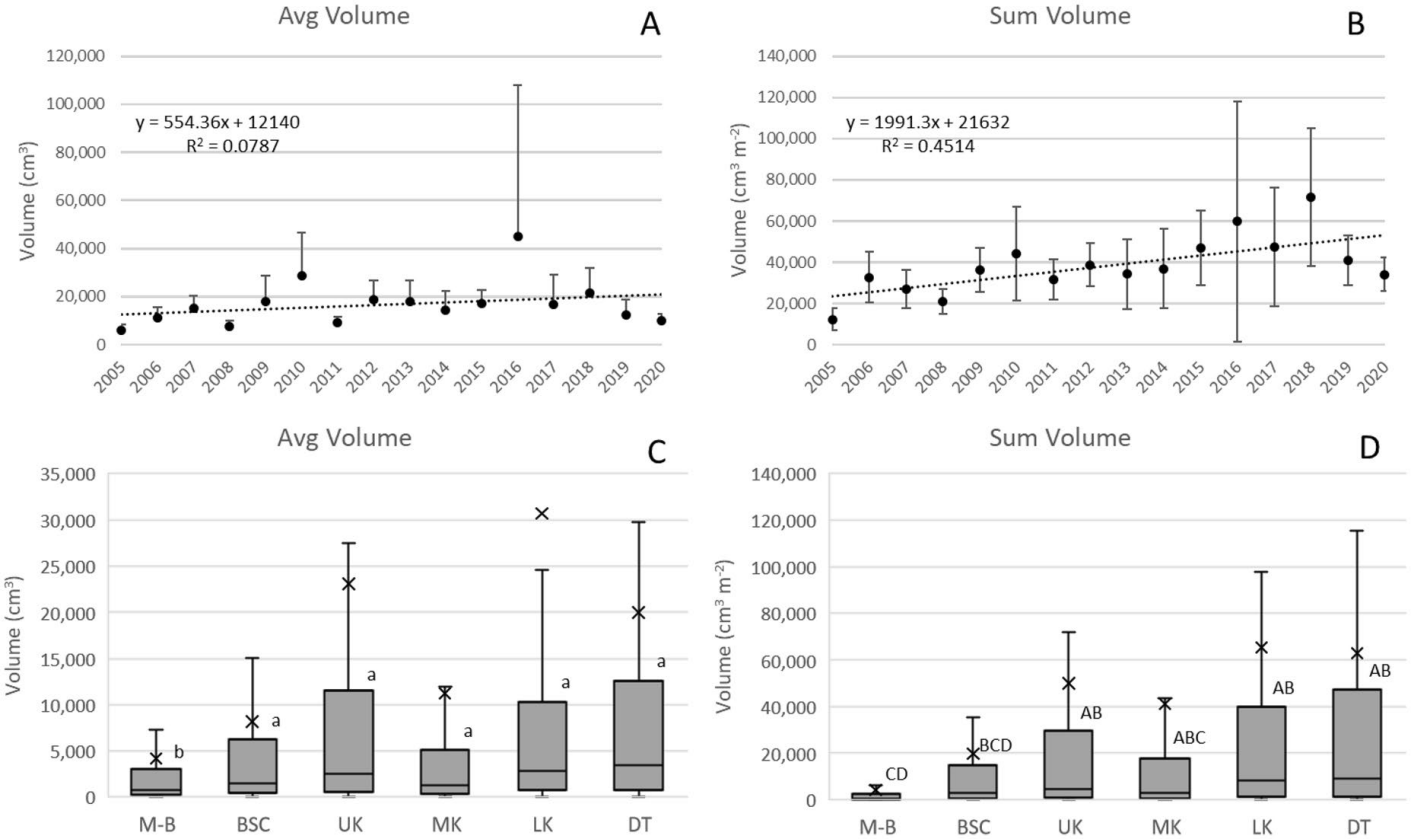
**A** and **B** Change over the duration of the survey (2005–2020) in Avg Vol and ΣVol, the latter increasing 1191 cm^3^ m^−2^ year^−1^ (*p* < 0.01). Vertical bars are 95% confidence intervals for each annual average; only upper confidence intervals are presented in **A**. Linear regressions are noted. **C** and **D** Differences in average and ΣVol across subregions. Boxplots show mean (x), median (crossbar) and data quartiles. Letters designate differences (*p* < 0.05) across subregions determined through analysis of variance using Tukey’s *post hoc *test criteria

There was no significant change in Avg Vol over the survey period (Fig. 6A) but ΣVol increased by 1991 cm^3^ m^−2^ year^−1^ (*p* < 0.01, Fig. 6B). This increase was driven primarily by Ofav, Ssid, Mcav, and Oann, which increased a combined 1882 cm^3^ m^−2^ year^−1^ (*p* < 0.01). Subregion M-B had significantly smaller Avg Vol relative to all other subregions (Fig. 6D), and ΣVol was significantly less at M-B and greater at UK, LK, and DT. One transect surveyed at LK in 2016 could be considered an outlier; there were nine colonies in the transect, including 3 Oann with Vol between 25 and 33 m^3^. This transect generated the high confidence intervals (CI) shown for 2016 (Fig. 5A, B), and if the transect is eliminated, the CIs shown are reduced by about half.

#### Regional trend

Colony density and size variables decreased with increasing latitude (northward) and increased with increasing longitude (westward; Table 4). Size variables, but not density, decreased with depth. In all cases, the trends are reported only for the ranges of latitude, longitude and depth recorded during the 16-year survey, and not outside these ranges.

**Table 4:** Increase or decrease ( −) in coral variables with each degree (°) latitude, degree longitude, and depth (ft). All values were significant at *p* < 0.001 except for ^a^ (*p* < 0.05) and ns (*p* > 0.05)

Variable	Latitude (°N)	Longitude (°W)	Depth (ft)
Density (n m^−2^)	− 1.4	0.8	ns
Avg H (cm)	− 1.1	0.5	− 0.1
Sum H (cm m^−2^)	− 12.7	7.5	− 0.1
Avg Fp (cm^2^)	− 3.4^a^	3.7	− 0.4
Sum Fp (cm^2^ m^−2^)	− 817.2	541.8	− 10.6
Avg Vol (cm^3^)	− 9665	6243	− 466.7
Sum Vol (cm^3^ m^−2^)	− 30,802	20,524	− 657.2
Avg SA (cm^2^)	− 385.3	238.8	− 24.7
Sum SA (cm^2^ m^−2^)	− 1956	1247	− 36.0

### Temporal variation within subregions

#### Martin-Broward (1502 transects; 13,116 colonies)

**Fig. 7:**
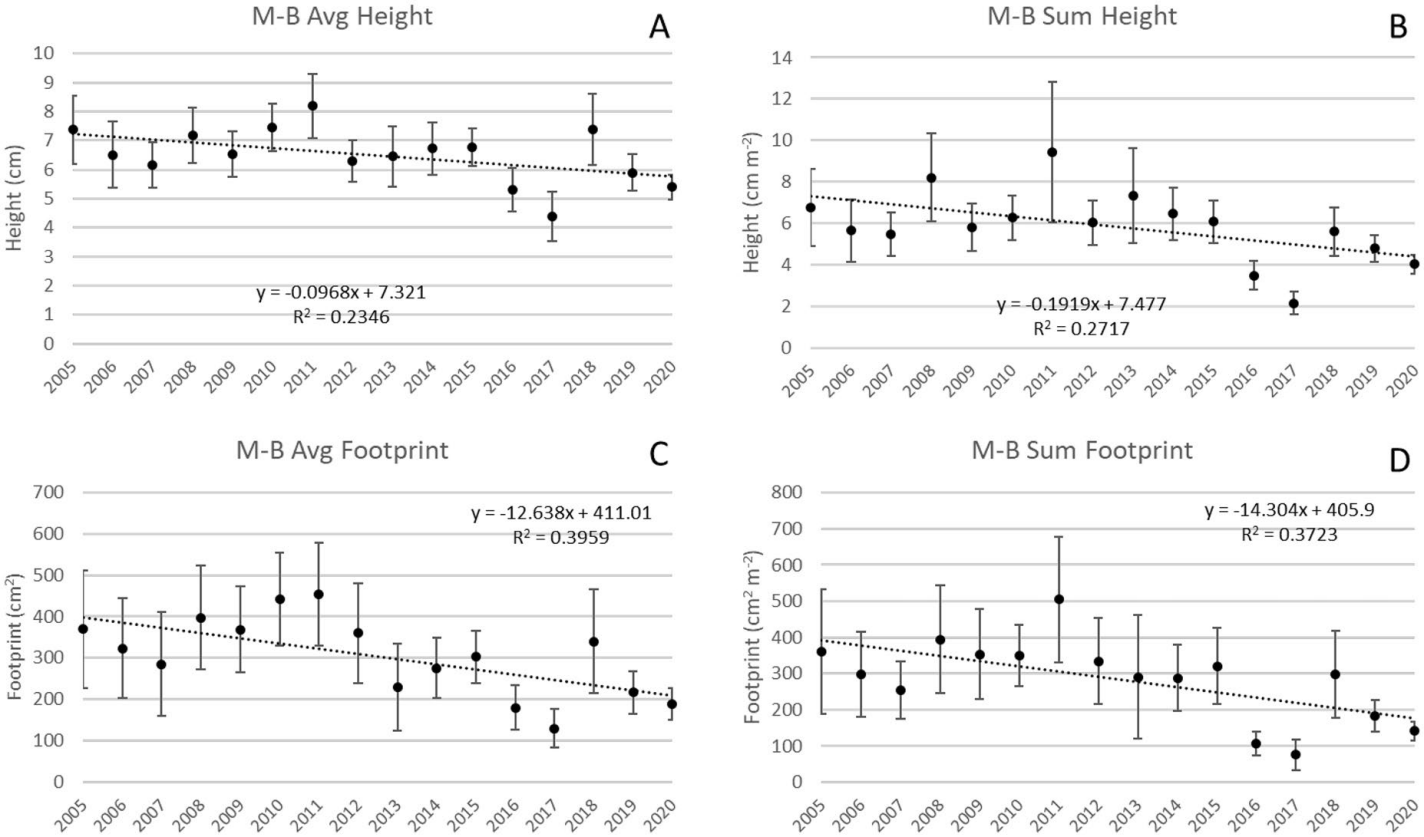
Changes at subregion M-B over the duration of the survey. **A** and **B** Avg H declined 0.1 cm year^−1^ and ΣH declined 0.2 cm m^−2^ year^−1^ (*p* < 0.05). **C** and **D** Avg Fp declined 12.6 cm^2^ year^−1^ and ΣFp declined 14.3 cm^2^ m^−2^ year^−1^ (*p* < 0.05). Vertical bars are 95% confidence limits for each annual average and linear regressions are noted

Avg H declined 0.1 cm year^−1^ and ΣH declined 0.19 cm m^−2^ year^−1^ (*p* < 0.05) at M-B during 2005–2020 (Fig. 7A, B). Avg Fp declined 12.7 cm^2^ year^−1^ and ΣFp declined 14 cm^2^ m^−2^ year^−1^ (*p* < 0.05, Fig. 7C). Other results that were not significant included a decrease in Avg SA (24.3 cm^2^ year^−1^, *p* = 0.08), a decline in ΣSA (27.7 cm^2^ m^−2^ year^−1^, *p* = 0.06), and a decline in colony density of 0.014 colonies year^−1^ (*p* = 0.19).

#### Biscayne (725 transects; 16,014 colonies)

There were no significant changes in density, size, or complexity of coral colonies at BSC during the 2005–2020 survey.

#### Upper Keys (950 transects; 21,640 colonies)

**Fig. 8:**
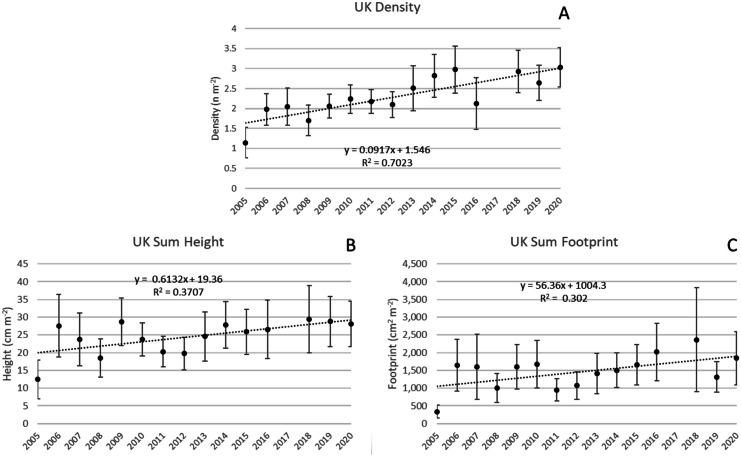
Changes at subregion UK over the duration of the survey. **A** Colony density increased 0.09 colonies year^−1^ (*p* < 0.001). **B** ΣH increased 0.6 cm m^−2^ year^−1^ (*p* < 0.05). **C** ΣFp increased 56.4 cm^2^ m^−2^ year^−1^. Vertical bars are 95% confidence limits for each annual average and linear regressions are noted

Colony density increased 0.09 colonies year^−1^ (*p* < 0.001) and ΣH increased 0.6 cm m^−2^ year^−1^ (*p* < 0.05, Fig. 8A, B) despite an apparent decrease in Avg H (0.15 cm year^−1^, *p* = 0.08). ΣFp increased 56.4 cm^2^ m^−2^ year^−1^ (*p* < 0.05; Fig. 8C).

#### Middle Keys (668 transects; 19,893 colonies)

There were no significant changes in density, size, or complexity of coral colonies at MK. However, density increased 0.07 colonies year^−1^ (*p* = 0.08) and there was relatively high variability in density from 2015 to 2020.

#### Lower Keys (1280 transects; 46,001 colonies)

**Fig. 9:**
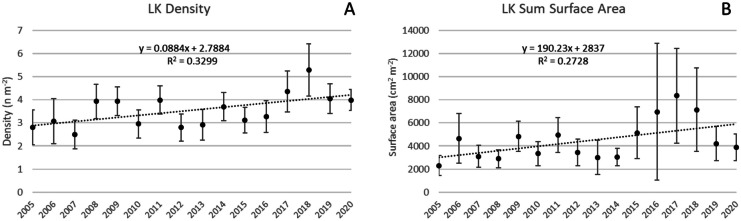
Changes at subregion LK during the survey period. **A** Colony density increased 0.09 colonies m^−2^ year^−1^ (*p* < 0.05). **B** ΣSA increased 190 cm^2^ m^−2^ year^−1^ (*p* < 0.05). Vertical bars are 95% confidence limits for each annual average and linear regressions are noted

Colony density increased 0.09 colonies m^−2^ year^−1^ (*p* < 0.05) at LK and ΣSA increased 190 cm^2^ m^−2^ year^−1^ (*p* < 0.05; Fig. 9). Other results include a decrease in Avg H (0.07 cm year^−1^, *p* = 0.08); an increase in ΣFp (74.5 cm^2^ m^−2^ year^−1^, *p* = 0.06); and an increase in ΣVol (5125 cm^3^ m^−2^ year^−1^, *p* = 0.05).

#### Dry Tortugas (891 transects; 29,893 colonies)

**Fig. 10:**
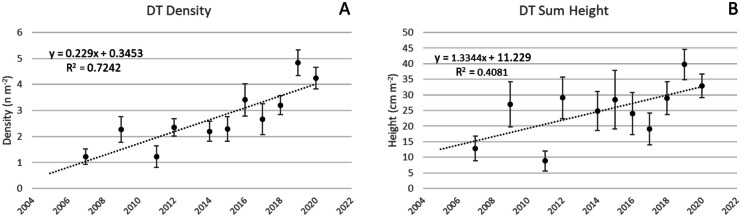
Changes at subregion DT during the survey period Colony density increased 0.23 colonies year^−1^ (*p* < 0.001) at DT and ΣH increased 1.3 cm m^−2^ year^−1^ (*p* < 0.05; Fig. 8) (**B**). Vertical bars are 95% confidence limits for each annual average and linear regressions are noted

Colony density increased 0.23 colonies year^−1^ (*p* < 0.001) at DT and ΣH increased 1.3 cm m^−2^ year^−1^ (*p* < 0.05; Fig. 10). Other results showed Avg H decreased 0.3 cm year^−1^ (*p* = 0.12).

## Discussion

The robust Disturbance Response Monitoring (DRM) dataset provided measurements to characterize coral colonies and reef structure across the Florida Reef Tract, which is among the largest in the world and extends roughly 360 miles (580 km) from Martin County in the northeast to Dry Tortugas in the southwest. This broad geographic region has substantial variation in climate, weather patterns, habitat, currents, human population, and other environmental factors that might influence coral size and distribution. The DRM program was initiated to document effects of high-temperature bleaching events. Since 2005, the program has accumulated a level of demographic information on coral colonies that is unsurpassed, including species and dimensions for over 150,000 colonies and with more added annually. Although ancillary to the purpose of the program, and despite the potential bias of sampling variability imposed over 16 years by weather events and resource availability, the DRM dataset provided a unique opportunity to examine spatial and temporal changes in stony coral colony species, abundance, size, and complexity.

Results from the survey showed colony density was greater at southwestern subregions and increased overall from 2005 to 2020, largely due to increased abundance of three physically small hemispheric species—Ssid, Past, and Sint. Increases in density across survey years were found for UK, LK, and DT subregions, outweighing a decrease at M-B. With the addition of these small, hemispheric colonies, average size and complexity were found to decrease over time while the total (sums) increased. Likewise, the disparity between large and small colonies created a deceptive incongruity in colony size data: The greatest average sizes were recorded for the large species, but the greatest contribution to total size came from the small, numerically dominant species. Reef structure, consequently, has changed from taller and more complex to shorter and simpler configurations.

Dustan and Halas (1987) speculated that the loss of *Acropora palmata* in Carysfort Reef (Upper Keys) could herald a shift in community composition toward smaller colonial species. This surmise has been well-supported by subsequent studies along the Florida Keys and Caribbean Sea (Alvarez-Filip et al., 2011; Burman et al., 2012; Green et al., 2008; Perry et al., 2015) as larger colonies seem more vulnerable to bleaching and disease (Shenkar & Loya, 2005; Brandt, 2009) and smaller colonies generally reproduce more rapidly and appear more tolerant of environmental stresses (Alvarez-Filip et al., 2011; Darling et al., 2012). This has led to a hypothesis of ongoing “homogenization” (Burman et al., 2012; Toth et al., 2019), or the emerging dominance of a few fast-growing and stress-tolerant species. The dominant and increasing abundance of Ssid, Past, and Sint in the DRM data supports and extends this model. These three species combined for more than 60% of all coral colonies in the survey and were widely distributed across all subregions; they were found at 80% (Ssid), 70% (Past), and 60% (Sint) of all 6016 stations in the surveys. Moreover, they were found to be increasing annually by six new colonies in every 100 m^2^ seafloor. Larger and more complex species, such as Apal, Acer, Dcyl, Oann, Ofav, and Ofra were sparse and comprised less than 5% of the recorded colonies.

Colony height measurements in the DRM dataset only partly corroborate the hypothesis that reefs are “flattening” (Alvarez-Filip et al., 2009, 2011). The proportion of larger colonies and the average height of colonies declined during 2005–2020, but this was largely due to apparent recruitment of smaller colonies, especially Ssid, Past, and Sint. The density of the larger colonies did not change for heights > 10 cm or > 50 cm and increased slightly for those > 100 cm. Reef flattening, then, can be more specifically characterized as an increase in reefs with shorter colonies without any apparent loss of reefs with taller colonies.

Only 20% of colonies in the DRM survey had heights > 10 cm (H_>10_) and this percentage dropped to 1.3% for H_>50_ and 0.2% for H_>100_ colonies. Colony size is ecologically important because it contributes reef architecture (surface area) for fish and invertebrate habitat (Alvarez-Filip et al., 2009; Darling et al., 2012; van Woesik & Jordan-Garza, 2011). Data from the Flower Garden Banks (Gulf of Mexico) and Puerto Rico have shown colony height to be positively correlated with fish populations and, importantly, colonies ≤ 10 cm height appear to contribute little to that relationship (Fisher, 2023). If this is also true in the Florida Reef Tract, then only 2 of 10 colonies are providing fish habitat. The proportion of H_>10_ colonies in Florida (20%) is low compared to proportions documented at the Flower Garden Banks (40%) and Puerto Rico (25–30%) (Fisher, 2023). The proportion of H_>10_ colonies in the DRM data declined between 2005 and 2020 but, because the density of H_>10_ colonies was unchanged, this decline in proportion was likely created by additions of new smaller colonies rather than losses of larger existing colonies.

Location coordinates and depth records at each sampling location allowed detection of spatial trends. Greater abundance, size, and complexity were found for transects more southward, more westward and, except for density, in shallower waters (Table 4). Additionally, temporal trends differed by subregion. For example, the density of colonies increased region-wide, but the increase at DT was substantially greater (0.23 colonies year^−1^) than other subregions, and densities at M-B, BSC, and MK showed no significant temporal change. M-B also stands out because it was the only subregion to decline through time in both average and sum of size variables, specifically H and Fp. BSC and MK are notable because of their consistency; there were no significant changes in any variable during the survey period.

Substrate is a limiting factor for colonization in most marine habitats (Dahl, 1973). The amount of benthic substrate occupied by corals, referred to as coral cover, can be quantified using colony footprint (Fp = π*r*^2^). In the DRM survey data, the sum of Fp was 1223 cm^2^ m^−2^, or ~ 12% of the area surveyed. This calculation of Fp over-estimates the actual footprint area because it assumes that colony radius is ½ the maximum diameter along its entire periphery. Also, the Fp includes both live and dead portions of colonies, which is different than traditional estimates of live coral cover. However, this disparity can be minimized by combining Fp with the Live Surface Area Index, which is the relation of live to total surface area (Fisher, 2022). Live Surface Area (LSA) Index was 63.4% for the DRM survey (data not shown), resulting in a live coral cover estimate of 7.8%. For comparison, live coral cover reported from 1984 to 1992 ranged from 12 to 32% (Porter & Meier, 1992) and more recently (2005–2014) the Coral Reef Evaluation and Monitoring Program (CREMP, 2016) reported live coral cover in the Florida Keys as 7–8.5%. It appears that by combining Fp with the LSA Index, demographic surveys can be reasonably compared with historical live coral cover estimates.

Colony-based measurements can be used to generate estimates of human benefits (ecosystem services) derived from coral reefs. For example, fish density has been estimated from the cumulative colony height, or ΣH, using the relationship fish density (n m^−2^) = 0.013*ΣH (cm) + 0.858 (Fisher, 2023). Recognizing the extrapolation caveats highlighted in online resource SI-2 and applying the average ΣH = 20.7 cm m^−2^ across all surveys and subregions, this calculation yields 1.13 fish m^−2^ and extrapolates to potential habitat for over 283 million fish along the 251 km^2^ Florida Reef Tract. Carbon storage, another example, can be estimated by combining ΣVol (average = 39,081 cm^3^ m^−2^ CaCO_3_) with the average CaCO_3_ density for all species (e.g., 1.649 g cm^−3^; Hughes, 1987; Fisher, 2022). Again, with extrapolation caveats emphasized, this calculation results in 64,445 g m^−2^ CaCO_3_ in stony corals, or over 16 billion kg (17.8 million tons) of CaCO_3_ stored across the Florida Reef Tract. Relative values from each subregion can be estimated separately. Other translations of physical data might be useful for estimating reduction in wave energy for coastal storm protection (Sheppard et al., 2005) or attraction for snorkeling and diving tourism (Moberg & Folke, 1999).

This study provides a baseline for future comparison as high-temperature events and other environmental pressures continue to affect the reef tract. Because colony characteristics were the focus, geographic subregions were designated based on the need to balance colony numbers. The subregion approach was supported by similar trends found in non-segmented latitude and longitude analyses. But this represents only one potential application of the rich DRM database, and other subsets may be better suited for other purposes. Some studies have used portions of the dataset to examine changes in reef structure (Burman et al., 2012); others to track potential outcomes from Stony Coral Tissue Loss Disease (Muller et al., 2020). Other variables in the dataset, such as partial mortality on colonies or reef zone and habitat classifications, could be employed. A tighter focus on specific subregions or time periods may prove instructive, even if ancillary to the original purpose of the program and sampling design.

The demographic approach is substantively different than 2-dimensional coral cover assessment methods such as line intercept, point intercept, and videography (Hill & Wilkinson, 2004) often used in Florida and elsewhere (e.g., Burns, 1985; Loya, 1976; Porter & Meier, 1992). Smith et al. (2011) noted that 2-dimensional coral cover measurements provide information on the net *outcome* of population rate processes (recruitment, growth, survival) whereas colony size and density measurements additionally provide information on the rate processes. As examples, the potential for habitat provision depends on measures of height and 3-dimensional colony surface area; the potential for primary productivity, nutrient cycling, carbon sequestration, reproduction, and skeletal growth depends on live surface area; and the potential for erosion depends on exposed skeletal surfaces (morbidity). The additional information gained from demographic surveys can be vital to assessing stony coral resilience during periods of increasing anthropogenic stress.

## Conclusions

A broad regional coral demographic survey across the Florida Reef Tract showed colony density increasing and average colony size decreasing between 2005 and 2020. This finding probably results from recruitment of three relatively small species, *Siderastrea siderea*, *Porites astreoides*, and *Stephanocoenia intersepta*. The dominance and growing abundance of these three species support a hypothesis of shifting community composition from larger, more complex species to smaller, stress-tolerant species. However, this trend was not evident in all subregions; while considerably stronger at the DT subregion, there was no evidence for a trend at the northeastern subregions, M-B and BSC, which maintained stable or slightly declining densities. The data appear to support a hypothesis of reef flattening, but only through an increase in smaller colonies rather than the loss of larger colonies. Nonetheless, only 20% of colonies in the DRM survey had heights > 10 cm, a critical size for fish habitat, and this is lower than percentages for Puerto Rico (25–30%) and the Gulf of Mexico (40%). The study demonstrates key advantages for including demographic assessments with traditional coral cover in the strength and variety of structural indicators and the quantitative relationships to ecosystem goods and services.

### Supplementary Information

Below is the link to the electronic supplementary material.

Supplementary file1 (PDF 176 KB)

## Data Availability

Data used in this study are publicly available on the DRM web site.
