# Supplementary material for: Reef structure of the Florida Reef Tract for the period 2005–2020
Source: Environ Monit Assess. 2023 Sep 22;195(10):1242. doi: 10.1007/s10661-023-11819-0 (PMC10516784; doi:10.1007/s10661-023-11819-0)
Supplement: Supplementary file 1 — Supplementary file1 (PDF 176 KB) [file 10661_2023_11819_MOESM1_ESM.pdf]

Supplementary Information

Environmental Monitoring and Assessment

**Reef Structure of the Florida Reef Tract for the Period 2005-2020**

William S. Fisher

U.S. Environmental Protection Agency (Emeritus)

Office of Research and Development

Gulf Environmental Measurement and Modeling Division

1 Sabine Island Drive, Gulf Breeze FL 32561 USA

Fisher.william@epa.gov

*SI-1: Species distributions across subregions.* Acronyms are defined in text; blanks signify no colonies, and 0.00 signifies <0.01.

| | **M-B** | | **BSC** | | **UK** | | **MK** | | **LK** | | **DT** | |
|---|---|---|---|---|---|---|---|---|---|---|---|---|
| **Taxon** | n $m^{-2}$ | % | n $m^{-2}$ | % | n $m^{-2}$ | % | n $m^{-2}$ | % | n $m^{-2}$ | % | n $m^{-2}$ | % |
| Acer | 0.02 | 2.22 | 0.02 | 0.69 | 0.01 | 0.49 | 0.00 | 0.05 | 0.01 | 0.19 | 0.02 | 0.67 |
| Apal | | | 0.00 | 0.01 | 0.00 | 0.08 | 0.00 | 0.01 | 0.00 | 0.02 | 0.00 | 0.05 |
| Apro | | | | | | | | | | | 0.00 | 0.03 |
| Aaga | 0.01 | 1.43 | 0.21 | 9.65 | 0.28 | 12.23 | 0.14 | 4.71 | 0.10 | 2.79 | 0.12 | 3.57 |
| Afra | 0.00 | 0.30 | 0.00 | 0.08 | 0.00 | 0.13 | 0.00 | 0.02 | 0.00 | 0.01 | 0.00 | 0.13 |
| Agra | | | | | | | 0.00 | 0.01 | | | 0.00 | 0.00 |
| Ahum | 0.00 | 0.02 | 0.00 | 0.22 | 0.01 | 0.30 | 0.00 | 0.02 | 0.00 | 0.09 | 0.01 | 0.19 |
| Alam | 0.00 | 0.25 | 0.01 | 0.49 | 0.01 | 0.62 | 0.01 | 0.18 | 0.01 | 0.19 | 0.01 | 0.25 |
| Aten | | | | | 0.00 | 0.01 | | | | | | |
| Carb | | | | | 0.00 | 0.01 | 0.00 | 0.01 | 0.00 | 0.03 | 0.00 | 0.02 |
| Cnat | 0.00 | 0.57 | 0.01 | 0.47 | 0.01 | 0.54 | 0.05 | 1.82 | 0.09 | 2.64 | 0.06 | 1.68 |
| Dcyl | | | 0.00 | 0.01 | | | | | 0.00 | 0.02 | | |
| Dsto | 0.03 | 3.16 | 0.13 | 5.89 | 0.08 | 3.65 | 0.10 | 3.41 | 0.09 | 2.62 | 0.04 | 1.20 |
| Dlab | 0.00 | 0.21 | 0.02 | 0.78 | 0.02 | 0.82 | 0.02 | 0.64 | 0.02 | 0.45 | 0.02 | 0.50 |
| Efas | 0.00 | 0.34 | 0.02 | 0.79 | 0.01 | 0.51 | 0.01 | 0.45 | 0.03 | 0.70 | 0.02 | 0.66 |
| Ffra | 0.00 | 0.05 | 0.00 | 0.11 | 0.00 | 0.05 | 0.00 | 0.10 | 0.00 | 0.10 | 0.00 | 0.02 |
| Hcuc | 0.00 | 0.01 | 0.00 | 0.07 | 0.01 | 0.25 | 0.00 | 0.02 | 0.00 | 0.04 | 0.00 | 0.11 |
| Irig | 0.00 | 0.01 | 0.00 | 0.02 | | | 0.00 | 0.01 | 0.00 | 0.01 | 0.00 | 0.00 |
| Isin | 0.00 | 0.03 | 0.00 | 0.02 | 0.00 | 0.00 | 0.00 | 0.04 | 0.00 | 0.06 | 0.00 | 0.02 |
| Maur | 0.01 | 1.28 | 0.00 | 0.02 | 0.00 | 0.05 | 0.00 | 0.07 | 0.00 | 0.05 | 0.01 | 0.18 |
| Mdec | 0.01 | 0.99 | 0.00 | 0.20 | 0.01 | 0.32 | 0.01 | 0.30 | 0.01 | 0.23 | 0.05 | 1.50 |
| Mfor | 0.00 | 0.01 | 0.00 | 0.04 | 0.00 | 0.08 | 0.00 | 0.02 | 0.00 | 0.03 | 0.00 | 0.02 |
| Msen | | | | | | | | | | | 0.00 | 0.02 |
| Mare | 0.00 | 0.03 | 0.00 | 0.01 | 0.00 | 0.07 | 0.00 | 0.14 | 0.00 | 0.10 | 0.01 | 0.19 |
| Mjac | | | 0.00 | 0.01 | | | 0.00 | 0.01 | | | 0.00 | 0.00 |
| Mmea | 0.03 | 3.25 | 0.02 | 0.98 | 0.01 | 0.46 | 0.01 | 0.45 | 0.02 | 0.55 | 0.04 | 1.11 |
| Mcav | 0.12 | 13.53 | 0.07 | 3.35 | 0.06 | 2.45 | 0.20 | 6.56 | 0.23 | 6.41 | 0.40 | 12.06 |
| Mang | 0.00 | 0.05 | 0.00 | 0.02 | 0.00 | 0.01 | 0.00 | 0.09 | 0.01 | 0.15 | 0.01 | 0.18 |
| Mali | 0.00 | 0.05 | 0.00 | 0.02 | 0.00 | 0.02 | 0.00 | 0.03 | 0.00 | 0.05 | 0.01 | 0.31 |
| Mfer | 0.00 | 0.01 | | | | | 0.00 | 0.01 | 0.00 | 0.01 | 0.00 | 0.04 |
| Mlam | 0.00 | 0.01 | 0.00 | 0.01 | 0.00 | 0.02 | 0.00 | 0.01 | 0.00 | 0.03 | 0.00 | 0.04 |
| Odif | 0.00 | 0.40 | 0.00 | 0.02 | 0.00 | 0.00 | 0.00 | 0.03 | 0.00 | 0.10 | 0.02 | 0.45 |
| Oann | 0.00 | 0.14 | 0.02 | 0.74 | 0.02 | 1.06 | 0.01 | 0.37 | 0.02 | 0.67 | 0.01 | 0.15 |
| Ofav | 0.01 | 0.66 | 0.03 | 1.51 | 0.05 | 2.31 | 0.05 | 1.51 | 0.08 | 2.25 | 0.09 | 2.63 |
| Ofra | 0.00 | 0.13 | 0.01 | 0.54 | 0.01 | 0.55 | 0.01 | 0.39 | 0.02 | 0.43 | 0.09 | 2.73 |
| Past | 0.19 | 21.51 | 0.40 | 18.27 | 0.39 | 17.11 | 0.45 | 15.04 | 0.53 | 14.61 | 0.64 | 19.18 |
| Pbra | 0.00 | 0.00 | 0.02 | 0.89 | 0.00 | 0.04 | 0.00 | 0.05 | 0.00 | 0.07 | 0.00 | 0.01 |
| Pdiv | 0.01 | 0.73 | 0.01 | 0.60 | 0.06 | 2.46 | 0.03 | 1.08 | 0.02 | 0.59 | 0.05 | 1.52 |
| Pfur | 0.00 | 0.02 | 0.04 | 1.90 | 0.04 | 1.71 | 0.02 | 0.67 | 0.02 | 0.47 | 0.04 | 1.18 |
| Ppor | 0.03 | 3.91 | 0.19 | 8.79 | 0.24 | 10.72 | 0.13 | 4.21 | 0.08 | 2.15 | 0.14 | 4.20 |
| Pcli | 0.01 | 1.56 | 0.02 | 0.75 | 0.01 | 0.58 | 0.01 | 0.38 | 0.01 | 0.26 | 0.01 | 0.44 |
| Pstr | 0.01 | 1.33 | 0.02 | 0.95 | 0.01 | 0.61 | 0.03 | 1.03 | 0.03 | 0.96 | 0.07 | 1.99 |
| Scub | 0.00 | 0.02 | 0.00 | 0.01 | 0.00 | 0.01 | 0.00 | 0.01 | 0.00 | 0.00 | 0.00 | 0.10 |
| Slac | | | | | | | | | 0.00 | 0.00 | 0.00 | 0.01 |
| Srad | 0.04 | 4.92 | 0.11 | 4.95 | 0.05 | 2.32 | 0.06 | 1.91 | 0.10 | 2.80 | 0.04 | 1.18 |
| Ssid | 0.15 | 17.46 | 0.65 | 29.35 | 0.72 | 31.41 | 1.12 | 37.58 | 1.32 | 36.78 | 0.91 | 27.04 |
| Sbou | 0.03 | 3.75 | 0.03 | 1.26 | 0.03 | 1.47 | 0.03 | 0.97 | 0.04 | 1.11 | 0.01 | 0.40 |
| Shya | 0.00 | 0.12 | 0.00 | 0.03 | 0.01 | 0.32 | 0.00 | 0.14 | 0.00 | 0.09 | 0.00 | 0.01 |
| Sint | 0.14 | 15.55 | 0.12 | 5.47 | 0.09 | 4.12 | 0.46 | 15.50 | 0.69 | 19.07 | 0.40 | 12.05 |
| **Total** | **0.87** | **100.00** | **2.21** | **100.00** | **2.28** | **100.00** | **2.98** | **100.00** | **3.59** | **100.00** | **3.35** | **100.00** |

*SI-2: Extrapolation of regional data to estimate selected ecosystem services.* Over the 16-yr DRM survey period examined, 6,016 10-$m^2$ transects were surveyed (0.06016 $km^2$), or roughly 0.024% of the targeted 251-$km^2$ Florida Reef Tract. Using relevant colony characteristics, simple extrapolation from the surveyed area to the entire tract can provide coarse estimates of several ecosystem services (Table SI-2). Several important caveats have bearing on these extrapolations: 1) corals are not equally or evenly distributed across all zones and habitats; 2) different zones and habitats are not considered in this analysis; and 3) the data reflect only 0.024% of the extrapolated area. Nonetheless, corals provide ecosystem services that should be considered in assessments. In this extrapolation, there are over 600 million colonies in the Florida Reef Tract, largely comprised of Ssid (190 million), Past (103 million) and Sint (80 million). The region provides 51 million m colony height, 30 million $m^2$ footprint, 72 million $m^2$ surface area, 45 million $m^2$ live surface area and roughly 10 million $m^3$. These dimensions are related to a variety of ecosystem services, including fisheries, storm protection, carbon sequestration and storage, and tourism and recreation.

**Table SI-2.** Summary data from survey periods 2005-2020 translated to regional estimates of colony density, height, footprint, volume and surface area. Normalized values were multiplied by 251 $km^2$, which is the estimated area of the Florida reef tract targeted by the surveys (Smith et al., 2011).

| Attribute | Result | Conversion | Regional Estimate |
|---|---|---|---|
| Colony Density | 2.44 colonies $m^{-2}$ | 2,440,000 colonies $km^{-2}$ | 612,440,000 colonies |
| Colony Height | 20.7 cm $m^{-2}$ | 207,000 m $km^{-2}$ | 51,057,000 m |
| Colony Footprint | 1,223.4 $cm^2$ $m^{-2}$ | 122,340 $m^2$ $km^{-2}$ | 30,707,340 $m^2$ |
| Colony Volume | 39,080.7 $cm^3$ $m^{-2}$ | 39,080.7 $m^3$ $km^{-2}$ | 9,809,256 $m^3$ |
| Colony Surface Area | 2,897.0 $cm^2$ $m^{-2}$ | 289,700 $m^2$ $km^{-2}$ | 72,714,700 $m^2$ |
